# Geoprogrammatic disparities in the performance of tuberculosis indicators in the homeless population in Brazil: an ecological approach

**DOI:** 10.1590/1980-549720230048.2

**Published:** 2023-10-27

**Authors:** Gabriel Pavinati, Lucas Vinícius de Lima, Cremilde Aparecida Trindade Radovanovic, Gabriela Tavares Magnabosco

**Affiliations:** I Universidade Estadual de Maringá, Programa de Pós-Graduação em Enfermagem - Maringá (PR), Brasil.

**Keywords:** III-Housed persons, Tuberculosis, Health status indicators, Spatial analysis, Ecological studies, Pessoas em situação de rua, Tuberculose, Indicadores básicos de saúde, Análise espacial, Estudos ecológicos

## Abstract

**Objective:**

To analyze the performance and spatial distribution of tuberculosis control indicators in the homeless population in Brazil.

**Methods:**

Ecological study, which had the regions and federal units of Brazil as the unit of analysis. The indicators considered, referring to the period from 2015 to 2021, were: proportion of HIV testing, proportion of tuberculosis-HIV co-infection, proportion of directly observed treatment, and proportion of outcomes (cure, treatment abandonment and death). The calculation was performed on each ecological unit, as recommended by the Ministry of Health. For the production of geographic figures, the technique of natural breaks was used.

**Results:**

It was identified that people living on the streets had: low HIV testing, especially in Pará (71.7%); high proportion of tuberculosis-HIV coinfection, especially in Rio Grande do Sul (39.9%); and unsatisfactory implementation of directly observed treatment, mainly in Paraíba (7.7%). With regard to outcomes, there was a high rate of treatment abandonment, with a higher proportion in Roraima (52.9%), and a high number of deaths, with an emphasis on Mato Grosso do Sul (23.1%), which also recorded the worst cure rate (28.7%).

**Conclusion:**

There was evidence of poor performance of tuberculosis control indicators in homeless people, with heterogeneous distribution between states and regions of the country, and it is clear that most of them had insufficient results. These data raise the persistence of difficulties and challenges inherent to the implementation of tuberculosis control strategies for this population in the national territory.

## RESUMO

**Objetivo:**

Analisar o desempenho e a distribuição espacial de indicadores de controle da tuberculose na população em situação de rua no Brasil.

**Métodos:**

Estudo ecológico, que teve como unidade de análise as regiões e as unidades federadas do Brasil. Os indicadores considerados, referentes ao período de 2015 a 2021, foram: proporção de testagem para HIV, proporção de coinfecção tuberculose-HIV, proporção de realização do tratamento diretamente observado e proporção dos desfechos (cura, abandono do tratamento e óbito). O cálculo foi efetuado sobre cada unidade ecológica, conforme recomendações do Ministério da Saúde. Para a produção das figuras geográficas, utilizou-se a técnica de quebras naturais.

**Resultados:**

Identificou-se que as pessoas em situação de rua apresentaram: baixa testagem para HIV, com destaque para o Pará (71,7%); alta proporção de coinfecção tuberculose-HIV, especialmente no Rio Grande do Sul (39,9%); e implementação insatisfatória do tratamento diretamente observado, principalmente na Paraíba (7,7%). No que se refere aos desfechos, verificou-se elevado abandono do tratamento, com maior proporção em Roraima (52,9%), e alto número de óbitos, com ênfase para o Mato Grosso do Sul (23,1%), que também registrou a pior proporção de cura (28,7%).

**Conclusão:**

Evidenciou-se baixo desempenho dos indicadores de controle da tuberculose nas pessoas em situação de rua, com distribuição heterogênea entre os estados e as regiões do país, sendo notório que a maioria deles teve resultados insuficientes. Esses dados suscitam a persistência de dificuldades e desafios inerentes à implementação das estratégias de controle da tuberculose para essa população no território nacional.

## INTRODUCTION

Tuberculosis (TB) still represents an important global public health problem^1^ ^,^ ^2^, and, with the advent of the COVID-19 pandemic, it is considered that there has been a setback in the advances obtained so far in controlling the disease, especially due to the repercussions caused by the reorganization of services and redirection of efforts to face the pandemic^1^. In 2020, there was an 18% reduction, compared to 2019, in the number of people newly diagnosed with TB worldwide^1^.

In the international scenario, the existence of goals for the end of TB as a public health problem, agreed between member countries of the United Nations, stands out. These targets seek to reduce the incidence of TB by 90% and deaths from the disease by 95% by 2035, compared to 2015 data^1^. To this end, the goals are: integrated and person-centered care actions, building support systems, strengthening research, and promoting equitable access to available technologies^1^.

Brazil is one of the countries with the highest TB burden, mainly due to the existing social and economic disparities between regions and states in the country, which also interfere with health indicators^3^. In this sense, boosting knowledge and debate about TB, which is strongly related to inequities and the social determination of the health-disease process, is essential for achieving the elimination goals agreed globally and adopted in the national context^4^.

According to data from the Brazilian Ministry of Health, there was a decrease in case notification during the COVID-19 pandemic period, from an incidence coefficient of 37.1 cases per 100,000 inhabitants in 2019 to 32.0 per 100,000 inhabitants in 2021^2^. Parallel to the reduction in incidence, there was a decrease in the proportion of cures, especially among TB cases in the homeless population (HLP), whose values went from 39.9% in 2018 to 33.0% in 2020^2^.

Studies carried out at the national level have shown unsatisfactory performance in terms of TB management and control in most of the assessed municipalities^5^ ^,^ ^6^. This scenario of programmatic weaknesses becomes even more complex when considering the catastrophic costs and socioeconomic consequences resulting from the illness and treatment for TB, such as unemployment, work incapacity, loss of income and social exclusion^7^, which make it difficult to eliminate the disease in Brazil.

With regard to HLP, there is a risk of illness due to TB increased around 56 times compared to the general population^8^. In addition, these people are more susceptible to unfavorable outcomes of treatment for the disease, especially those related to loss of follow-up and death^4^ ^,^ ^8^. This is a group that experiences situations of health and food insecurity, violence and prejudice in their daily lives, impairing willingness and adherence to care^9^ ^,^ ^10^.

Considering that HLP constitute a priority group for TB control and that socioeconomic and programmatic barriers related to the management of the disease in the country still persist, which directly and indirectly influence the monitoring of affected people, the present study aimed to: analyze the performance and spatial distribution of TB control indicators in HLP in Brazil.

## METHODS

This was a descriptive geospatial ecological study, whose unit of analysis were the geographic regions and states of Brazil. Data came from the Notifiable Diseases Information System (*Sistema de Informação de Agravos de Notificação* - SINAN), accessed in October 2022 by the Department of Informatics of the Unified Health System (*Departamento de Informática do Sistema Único de Saúde* - DATASUS). This research followed the recommendations of the Reporting of Studies Conducted using Observational Routinely-Collected Health Data (RECORD)^11^.

SINAN is a decentralized system for Brazilian municipalities that enables the continuous consolidation of data related to compulsorily notifiable diseases in Brazil, being the main source of information used by surveillance for the planning of health actions^12^. Specifically, regarding TB, only confirmed cases are commonly notified by health professionals at any level of care, which are then followed up for about six months until treatment is completed^12^.

Brazil has 203,062,512 inhabitants^13^ and presents inequalities in income distribution, with a Gini coefficient of 51.0 in 2022^14^. The country is segmented into 27 states, which are organized into five regions: North, Northeast, South, Southeast, and Central West^13^. Despite the lack of official HLP counts in Brazil, the Institute of Applied Economic Research (*Instituto de Pesquisa Econômica Aplicada* - IPEA) estimated that 221,869 people were in this situation in 2020, an increase of 140% compared to 2012^15^.

As the population of this study, TB cases registered in adults aged 20 to 59 years were considered. To delimit the cases in people living on the streets, the variable HLP (yes; no; ignored) from SINAN was used, including only cases that had this variable marked “yes”. Also, the period from 2015 to 2021 was delimited, considering that 2015 was the year of insertion of this variable in the TB notification form^12^.

Among the indicators for monitoring TB in Brazil^2^ ^,^ ^15^, those necessary to explain aspects of follow-up and the outcome of cases were selected: proportion of new cases tested for human immunodeficiency virus (HIV); proportion of new cases with TB-HIV coinfection; proportion of new cases who underwent directly observed treatment (DOT); proportion of new cured cases; proportion of new cases that abandoned treatment; and proportion of new cases that evolved to death.

The concept adopted by the Ministry of Health was assumed as a new case, which considers people who never underwent treatment or who did so for less than 30 days. In addition, cases in which this information is non-existent (entry in SINAN as “unknown” or “post-death”) are also considered as new^2^ ^,^ ^16^. When referring only to TB cases, records with any entry in the system were considered (new case; relapse; re-entry after abandonment; unknown; transfer; post-death; ignored).

Data were downloaded by year of diagnosis and place of residence (region and state), considering the variables necessary for the construction of indicators, namely: HIV testing (positive; negative; in progress; not performed; ignored); completion of DOT (yes; no; ignored); and termination situation (cure; abandonment; death from TB; death from other causes; transfer; change of diagnosis; drug-resistant TB; change of regimen; bankruptcy; primary abandonment; ignored).

Calculation of TB control indicators in HLP was performed for each region and state, based on the amount of data recorded in the year, according to the formulas recommended by the Ministry of Health (Chart 1)^2^ ^,^ ^16^. Given the lack of a HLP census denominator for each ecological unit, the proportion was used as a measure. First, the historical series was elaborated in order to visualize the behavior of the indicators from 2015 to 2021.

**Chart 1.** Description of the performance evaluation indicators for tuberculosis control and the respective formulas used for calculation in this study^2^ ^,^ ^16^.

Indicator	Formula
Proportion of tuberculosis cases tested for HIV	Numerator: new cases* with HIV test carried out^†^ in a given period. Denominator: new cases* in the same period and location. Multiplication factor: 100.
Proportion of cases of tuberculosis-HIV co-infection	Numerator: new cases* with a positive HIV test in a given period. Denominator: new cases* in the same period and location. Multiplication factor: 100.
Proportion of tuberculosis cases that underwent directly observed treatment	Numerator: new cases* with supervised treatment in a given period. Denominator: new cases* in the same period and location. Multiplication factor: 100.
Proportion of cases of tuberculosis with closure by cure	Numerator: new cases* closed due to cure, according to year of diagnosis. Denominator: new cases* in the same period and location. Multiplication factor: 100.
Proportion of tuberculosis cases with closure due to abandonment	Numerator: new cases* closed due to abandonment^‡^, according to year of diagnosis. Denominator: new cases* in the same period and location. Multiplication factor: 100.
Proportion of cases of tuberculosis with closure due to death	Numerator: new cases* closed by death^§^, according to year of diagnosis. Denominator: new cases* in the same period and location. Multiplication factor: 100.

HIV: human immunodeficiency virus; *type of entry: new case, unknown and post-death; ^†^HIV test result: positive and negative; ^‡^ending situation: abandonment and primary abandonment; ^§^closure situation: death from tuberculosis and other causes.

For the distribution of the performance of the indicators, the arithmetic mean of the data for the period from 2015 to 2019, years prior to the COVID-19 pandemic, was considered. This delimitation took into account the possible interference caused by the restructuring of health systems in the face of the sanitary exceptionality imposed by COVID-19, especially of surveillance services and information systems^17^, which may have altered the epidemiological reality of TB in the HLP.

Based on this, geographic figures were produced using the QGIS^®^ software, version 2.8, using the shapefile of the map of Brazil obtained from the Brazilian Institute of Geography and Statistics (*Instituto Brasileiro de Geografia e Estatística* - IBGE)^12^. For the spatial distribution, the technique of natural breaks was used, which allowed the visualization of arrangements in different categories, represented, in this case, by darker colors in states with greater proportions and lighter colors for places with smaller proportions.

With regard to ethical aspects, it is mentioned that this research, linked to the master’s thesis, was approved by the Ethics Committee in Research with Human Beings of Universidade Estadual de Maringá, as recommended by Resolution No. 466/2012 (opinion No. 5.721.740/2022). It should also be noted that, as these are aggregated and non-nominal data, whose access is in the public domain, the waiver of the informed consent was requested.

## RESULT S

**Figure 1.**
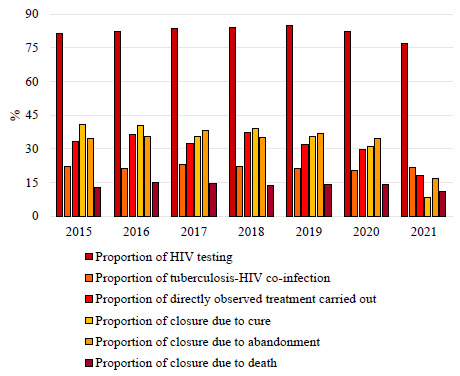
Proportion of indicators for monitoring and closing tuberculosis cases in the homeless population in Brazil from 2015 to 2021.

Between 2015 and 2021, 21,165 cases of TB were reported in the HLP across the country, of which 11,772 were registered as new cases and included in this study. It was observed, at the national level, that the indicators referring to the proportion of people on the streets tested for HIV (85.0% in 2019 to 76.9% in 2021), with TB-HIV co-infection (21.9% in 2019 to 20.4% in 2021) and who underwent DOT (32.1% in 2019 to 18.2% in 2021) showed a decrease (Figure 1).

HIV: human immunodeficiency virus.

Regarding the outcome of TB cases in homeless people, the proportions of cure (35.5% in 2019 to 8.4% in 2021), abandonment (36.9% in 2019 to 16.7% in 2021), and death (14.0% in 2019 to 11.1% in 2021) also showed a decline in the comparison between the year immediately preceding the pandemic and the second year of the pandemic, which denotes that the outcomes of these cases were concentrated in the closure categories not analyzed in this study (Figure 1).

In the analysis by region, HIV testing ranged from 77.0% in the North to 90.3% in the South. The proportion of TB-HIV co-infection ranged from 17.5% in the Central West to 34.8% in the South. As for the performance of the DOT, the proportions varied between 28.2% and 43.5% in the Northeast and North, respectively. Regarding the outcome, the cure rate ranged from 34.5% in the South to 42.7% in the North; abandonment between 32.1% in the North and 38.1% in the Southeast; and death from 11.0% in the Northeast to 16.6% in the Central West (Table 1).

**Table 1.** Number of new cases and proportion of indicators for monitoring and closing tuberculosis cases in the homeless population, by region and state of Brazil from 2015 to 2019.

Location	n	HIV*	TB-HIV^†^	DOT^‡^	Cure	Abandonment	Death
North region	452	77.0	20.1	43.5	42.7	32.1	11.3
Rondônia	42	76.2	9.5	26.2	50.0	35.7	2.4
Acre	19	84.2	0.0	63.2	73.7	10.5	15.8
Amazonas	162	79.0	27.8	9.9	38.3	38.3	17.3
Roraima	17	88.2	5.9	76.5	35.3	52.9	11.8
Pará	184	71.7	20.7	25.5	42.4	27.2	8.7
Amapá	9	77.8	0.0	55.6	55.6	22.2	0.0
Tocantins	19	94.7	15.8	47.4	36.8	26.3	5.3
Northeast region	1,410	80.7	21.8	28.2	36.8	33.0	11.0
Maranhão	136	88.2	20.6	20.6	38.2	42.6	14.0
Piauí	50	78.0	18.0	36.0	34.0	36.0	10.0
Ceará	355	81.7	22.5	46.5	36.1	37.5	11.0
Rio Grande do Norte	103	84.5	21.4	32.0	34.0	25.2	13.6
Paraíba	52	86.5	21.2	7.7	30.8	30.8	5.8
Pernambuco	269	75.5	21.2	22.7	37.2	25.3	13.4
Alagoas	80	83.8	26.3	10.0	38.8	36.3	2.5
Sergipe	75	86.7	18.7	34.7	36.0	41.3	10.7
Bahia	290	76.6	22.8	18.6	39.0	29.7	10.0
Southeast region	4,653	82.9	18.3	35.6	39.3	38.1	14.2
Minas Gerais	504	83.7	25.2	40.9	37.3	33.1	17.1
Espírito Santo	159	86.8	25.2	41.5	31.4	34.6	17.0
Rio de Janeiro	1,131	87.0	17.2	48.5	36.3	38.3	9.2
São Paulo	2,859	80.9	17.2	29.2	41.3	39.2	15.5
South region	1,453	90.3	34.8	39.0	34.5	34.1	16.5
Paraná	372	91.1	26.9	61.6	40.3	21.5	20.2
Santa Catarina	276	89.1	30.4	53.6	35.5	28.6	14.5
Rio Grande do Sul	805	90.3	39.9	23.5	31.4	41.7	15.5
Central West region	434	78.3	17.5	34.1	34.8	32.5	16.6
Mato Grosso do Sul	108	85.2	22.2	32.4	28.7	33.3	23.1
Mato Grosso	118	67.8	18.6	35.6	39.0	32.2	11.0
Goiás	156	79.5	10.9	34.0	37.2	33.3	17.9
Federal District	52	88.5	25.0	34.6	30.8	28.8	11.5
Brazil	8,402	83.3	21.8	36.1	38.0	36.0	14.0

HIV: human immunodeficiency virus; TB: tuberculosis; DOT: directly observed treatment. *testing for HIV; ^†^tuberculosis-HIV co-infection; ^‡^directly observed treatment carried out.

**Figure 2.**
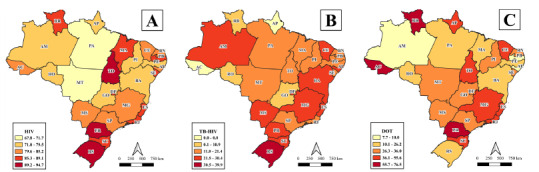
Spatial distribution of proportion indicators for HIV testing (A), tuberculosis-HIV co-infection (B) and performance of directly observed treatment (C) of tuberculosis cases in the homeless population, according to states of Brazil from 2015 to 2019.

HIV: human immunodeficiency virus; TB: tuberculosis; DOT: directly observed treatment.

The highest proportions of HIV testing were in Tocantins, Paraná, and Rio Grande do Sul (89.2-94.7%) and the lowest were in Mato Grosso and Pará (67.8-71.7%) (Figure 2A). With regard to TB-HIV co-infection, Rio Grande do Sul (39.9%) had the highest proportion, and Amapá and Acre did not register cases (Figure 2B). As for DOT, higher proportions were observed in Roraima, Acre, and Paraná (55.7-76.5%) and lower in Paraíba, Amazonas, and Alagoas (7.7-10.0%) (Figure 2C).

**Figure 3.**
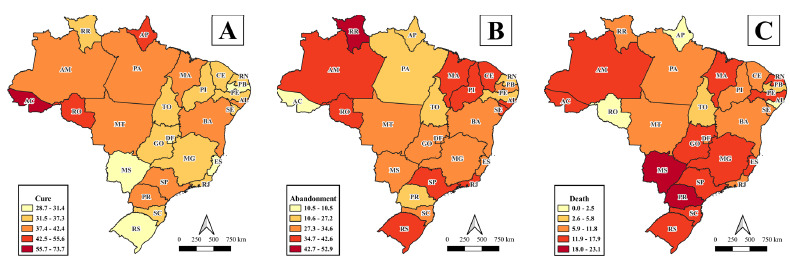
Spatial distribution of indicators of proportion of closure due to cure (A), abandonment (B) and death (C) of tuberculosis cases in the homeless population, according to federated units of Brazil from 2015 to 2019.

A higher proportion of cure was observed in Acre (73.7%) and lower ones in Mato Grosso do Sul, Paraíba, Espírito Santo, and Rio Grande do Sul (28.7-31.4%) (Figure 3A). The lowest dropout rate was identified in Acre (10.5%) and the highest in Roraima (52.9%) (Figure 3B). With regard to death, smaller proportions were observed in Amapá, Rondônia, and Alagoas (0.0-2.5%) and higher in Mato Grosso do Sul and Paraná (18.0-23.1%), respectively (Figure 3C).

## DISCUSSION

This study revealed poor performance of the follow-up and closure indicators of TB cases in the HLP. There was low HIV testing, a high proportion of individuals with TB-HIV co-infection and unsatisfactory implementation of DOT, with heterogeneous distribution between states and regions of the country. Regarding the outcomes of the cases, a high rate of treatment abandonment and death from TB and a low rate of cure were identified among homeless people.

TB control strategies, in addition to biological aspects, must be linked to social and structural issues^18^, which are sometimes marked by inequality and worse living conditions. Thus, it is necessary to broaden the understanding about the health-disease process related to TB, introducing indicators and markers that fully contemplate the aspects related to individuals, their contexts and the resources they have to face the disease^19^ ^,^ ^20^.

In Brazil, testing for HIV and other sexually transmitted infections (STIs) is recommended for all homeless people with TB^21^. However, it was found that, in this study, in most states, the proportion of people tested was less than 90%, with the exception of Tocantins, Paraná, and Rio Grande do Sul. This finding signals a warning to public authorities, since that TB is the most common cause of death in people living with HIV/acquired immunodeficiency syndrome (AIDS)^22^.

The proportion of TB-HIV co-infection, in turn, was high among HLP in all regions of the country, and the worst indicators were seen in the states of Rio Grande do Sul and Santa Catarina. These findings may be linked to the high circulation of HIV in these territories. Rio Grande do Sul and Santa Catarina had the second (21.8/100,000 inhabitants) and fourth (19.7/100,000 inhabitants) highest HIV detection rates in the country in 2021, respectively^23^.

Ecological studies carried out in Ethiopia identified that poor access to health care, abuse of psychoactive substances, low wealth index, and low adult literacy rate were important predictors for TB-HIV co-infection^24^ ^,^ ^25^. These factors can be commonly found in homeless people and vary between Brazilian states. Thus, they should be considered as potential explanatory factors for the findings of this study.

Although early diagnosis and timely treatment are essential to reduce morbidity and mortality among people with TB-HIV co-infection^22^, the results of this research pointed to the fragility of care for SP, which daily faces barriers in accessing health services^26^. Therefore, it is essential that there is integration between TB and HIV/AIDS control programs in order to reduce the strong presence of co-infection among this public in the country.

Low DOT was also evidenced; the proportion was less than 40% nationwide. This value is at odds with the recommendation that this treatment modality be offered to all homeless people^21^. These findings corroborate Brazilian research that showed the weak development and scarce implementation of DOT in other population groups, given structural and operational difficulties of health services^27^ ^,^ ^28^ ^,^ ^29^.

Performing DOT is associated with higher rates of cure and reduced loss to follow-up, which reinforces the effectiveness of this action as an approach to care management^27^. A retrospective cohort study carried out in Hunan, China, which included 306,860 people with TB, identified that complete supervision of treatment in health services, in relation to self-administration or partial supervision, was a factor associated with the successful outcome of cases^30^.

In Namibia, an interrupted time series study, which evaluated the use of DOT in the community, showed that this strategy increased the annual success rate of TB treatment by 12.9%^31^. In addition, a systematic review with meta-analysis also reported community DOT, an action to decentralize TB treatment from health units to the community, as a promising tool, especially in low-income countries with a high TB burden^32^.

An ecological study carried out in the state of São Paulo, Brazil, demonstrated that lower performance of DOT was associated with the formation of risk areas for the incidence of TB-HIV co-infection and treatment abandonment^33^. That said, it signals an alert for the states with the lowest rates of DOT implementation, such as Amazonas, Alagoas and Paraíba, regarding the need to strengthen this care tool at all points of the health care network, especially to the HLP.

In Brazil, it is recommended that cure indicators be greater than 90% and that abandonment and death rates be reduced to less than 5% of cases^34^. In this study, a great discrepancy between the observed and expected reality was evidenced. Similarly, descriptive research with data from SINAN and Cadastro Único (CadÚnico) identified that, in 2018, only 34.5% of the HLP with TB were cured, 32.6% of these people were lost to follow-up, and 7.5 % died from TB^35^.

In the international literature, the unfavorable outcome of TB in HLP has also been reported. For example, a cohort study carried out in Italy showed that being homeless increased by 3.23 times (95% confidence interval [95%CI] 2.58-4.54) the chance of death, failure or loss of follow-up^36^. In the United States of America, it was shown that the chance of death from HP was 2.26 times higher (95%CI 1.68-3.03) when compared to people with a fixed residence^37^.

A systematic review pointed out that TB in homeless people remains neglected worldwide, especially in developing countries given the burden of poverty and social inequality experienced^38^. This scenario drives the need for intersectoral actions aimed at serving this public in its entirety, since these people have complex life dynamics, which makes prevention, diagnosis, and completion of TB treatment difficult^35^.

In Brazil, in addition to the daily challenges faced by homeless people, there are numerous adversities imposed on services that make it difficult to implement the Tuberculosis Control Program (*Programa de Controle da Tuberculose* - PCT) and to improve disease indicators, such as: shortage of human resources, high professional turnover, fragmentation of care, professional unpreparedness in case management, lack of integration between coordination and health units, among others^39^.

Thus, the findings of this research confirm the position of extreme vulnerability in which homeless people find themselves, regardless of the geographic space they occupy. In general, all states in the country presented unsatisfactory operational indicators with regard to TB control. This reiterates that, in addition to social and individual issues, it is necessary to consider the important influence of programmatic aspects in the management of the disease.

It is noteworthy that this study has limitations, including: the possibility of incompleteness and erroneous completion of the notification forms that feed SINAN, which may have distorted the real TB indicators in the HLP. Furthermore, it is admitted as a limitation the incorporation of the variable for HLP in SINAN only in the first years of the historical series, which may have favored underreporting due to the adaptation period on the part of health professionals.

A previous study showed low completeness of the HLP field and treatment follow-up, such as performing the DOT; no update of the HIV test variable; and existence of records without closure. Therefore, findings based on these data may not faithfully represent the epidemiological scenario; however, SINAN is recognized as a source of information for planning and decision-making in health in the Brazilian context^13^.

In short, this geospatial ecological study showed that the performance of TB control indicators in the HLP showed heterogeneous distribution between states and regions of the country. It was also observed that most of the indicators had unsatisfactory performance in all locations, which expresses the existence of difficulties and reaffirms the complex challenges involved in the implementation of TB control actions for this population in the national territory.

In this sense, the importance of articulation and integration between the various social actors is recognized, such as health services, social assistance, public security and civil society, through interprofessional and interdisciplinary action^40^, with a view to offering comprehensive and continuous care to people living on the streets. Furthermore, there is an urgent need to qualify and implement new strategies aimed at the health care of the homeless, with a view to improving TB care and control.
